# Supplementary material for: TextFormats: Simplifying the definition and parsing of text formats in bioinformatics
Source: PLoS One. 2022 May 26;17(5):e0268910. doi: 10.1371/journal.pone.0268910 (PMC9135226; doi:10.1371/journal.pone.0268910)
Supplement: S1 Appendix — Examples of Python, Nim, Bash and C code using the TextFormats library for parsing a text format. (PDF) [file pone.0268910.s001.pdf]

S1 Appendix: Example code based on *TextFormats*

Giorgio Gonnella[1*]

1 Department of Bioinformatics (IMG), University of Göttingen, Göttingen, Germany

* E-mail: giorgio.gonnella@uni-goettingen.de (GG)

The following code exemplifies some of the basic functionality of the *TextFormats* library in the different supported programming and scripting languages (Python, Nim, Bash and C).

The examples assume that the file `cigars.tf.yaml` contains the following definitions of CIGAR operation and CIGAR string:

```
datatypes:
  cigar_op: {composed_of: [length: {unsigned_integer: {min: 1}},
                           code: {values: [M,I,D]}]}
  cigar_str: {list_of: cigar_op, minlength: 0}
```

1 Python

```
from textformats import Specification

SPECIFICATION = "cigars.tf.yaml"
DATATYPE_NAME = "cigar_str"
TEXT_REPRESENTATION = "100M10D"
DECODED_JSON = "[{"length":100,"code":"M"},"+\
                "{"length":10,"code":"D"}]"

# (1) get datatype definition
spec = Specification(SPECIFICATION)
datatype = spec[DATATYPE_NAME]

# (2) decode text representation and print decoded value as JSON
print("Decoded value: " +\
      datatype.decode(TEXT_REPRESENTATION, True))

# (3) encode decoded value to text representation
print("Text representation: " +\
      datatype.encode(DECODED_JSON, True))
```

2 Nim

```
import textformats

const
  SPECIFICATION = "cigars.tf.yaml"
  DATATYPE_NAME = "cigar_str"
  TEXT_REPRESENTATION = "100M10D"
  DECODED_JSON = [{\"length\":100,\"code\":\"M\"}," &
                  {\"length\":10,\"code\":\"D\"}]"

# (1) get datatype definition
let
  spec = specification_from_file(SPECIFICATION)
  datatype = spec.get_definition(DATATYPE_NAME)

# (2) decode text representation and print decoded value as JSON
echo("Decoded value: " &
     TEXT_REPRESENTATION.decode(datatype))

# (3) encode decoded value to text representation
echo("Encoded value: " &
     parse_json(DECODED_JSON).encode(datatype))
```

3 Bash

```
SPECIFICATION="cigars.tf.yaml"
DATATYPE_NAME="cigar_str"
TEXT_REPRESENTATION="100M10D"
DECODED_JSON="[{\"length\":100,\"code\":\"M\"},"
DECODED_JSON+="{\"length\":10,\"code\":\"D\"}]"

# (1) decode text representation and print decoded value as JSON
echo -n "Decoded value: "
tf_decode string -s $SPECIFICATION \
                 -t $DATATYPE_NAME \
                 -e $TEXT_REPRESENTATION

# (2) encode decoded value to text representation
echo -n "Encoded value: "
tf_encode json -s $SPECIFICATION \
               -t $DATATYPE_NAME \
               -d $DECODED_JSON
```

4 C

```
#include "textformats_c.h"
#include <stdio.h>
#include <assert.h>

#define SPECIFICATION "cigars.tf.yaml"
#define DATATYPE_NAME "cigar_str"
#define TEXT_REPRESENTATION "100M10D"
#define DECODED_JSON  "[{\"length\":100,\"code\":\"M\"}," \
                       "{\"length\":10,\"code\":\"D\"}]"

int main(void) {
  /* (1) setup */
  NimMain();
  tf_quit_on_err = true;

  /* (2) get datatype definition */
  Specification *spec =
    tf_specification_from_file(SPECIFICATION);
  DatatypeDefinition *datatype =
    tf_get_definition(spec, DATATYPE_NAME);

  /* (3) decode text representation */
  char *encoded = TEXT_REPRESENTATION;
  JsonNode *node = tf_decode(encoded, datatype);
  printf("Decoded value: %s\n", jsonnode_to_string(node));

  /* (4) encode value to text representation */
  char *decoded = DECODED_JSON;
  printf("Encoded value: %s\n",
         tf_encode_json(DECODED_JSON, datatype));

  /* (5) cleanup */
  delete_jsonnode(node);
  tf_delete_specification(spec);
  tf_delete_definition(datatype);

  return 0;
}
```
