# Supplementary material for: TextFormats: Simplifying the definition and parsing of text formats in bioinformatics
Source: PLoS One. 2022 May 26;17(5):e0268910. doi: 10.1371/journal.pone.0268910 (PMC9135226; doi:10.1371/journal.pone.0268910)
Supplement: S2 Appendix — Comparisons of the performance and features of the currently available regular expression libraries for the Nim programming language: re, nre, regex and nregex. (PDF) [file pone.0268910.s002.pdf]

S2 Appendix: Comparison of the available regular expression libraries for Nim

Giorgio Gonnella[1*]

1 Department of Bioinformatics (IMG), University of Göttingen, Göttingen, Germany

* E-mail: giorgio.gonnella@uni-goettingen.de (GG)

Four regular expression libraries were available for Nim: two in the standard library (re and nre) and two as nimble packages (regex and nregex).

The packages were tested on a file containing 100 thousand randomly generated CIGAR strings (one per line), with an average of 100 operations each (one of M, D, I or P). Thereby, four different operations were tested:

- Validate the CIGAR string, using the regular expression `(?:+̣[MDIP])+`. No groups were captured in this operation.
- Validate the CIGAR string and split it into single CIGAR operations using the regular expression `(?:+̣)(?:[MDIP])` iteratively. No groups were captured in this operation.
- Validate the CIGAR string, split it into single CIGAR operations and access length and operation code via capturing groups, using the regular expression `(+̣)([MDIP])` iteratively.
- Validate the CIGAR string and split it into single CIGAR operations, and access length and operation code via named capturing groups, using the regular expression `(?P<length>+̣)(?P<code>[MDIP])` iteratively.

The running time was measured as real time measured by GNU *time*. The values given in the table are the average of 3 run. The benchmarks were run on a Linux workstation (CPU Intel i5-4570 3.20GHz, 8 Gb RAM, Linux OpenSuse 15.1).

Since TextFormats requires to parse the single components of text representations, capturing groups are used. The results show that, when using groups, the `regex` library is faster than the standard library `re` and `nre` and than the `nregex` Nimble library.

Furthermore, the `regex` library allows compiling the regular expressions before use, which is useful in *TextFormats*, where the regular expression for a datatype is often used multiple time. It also allows for capturing multiple instances of groups (e.g. `()̣+`), which is very useful e.g. when parsing unordered list of equivalent elements.

Table 1: **API functions in Nim/C/Python and CLI commands for performing the most important operations implemented by *TextFormats***

Operation	Nim Library	Real time (s)
Validate the CIGAR string	re	0.5
	nre	0.5
	nregex	0.6
	regex	1.0
Validate and split into CIGAR operations	re	1.0
	nre	6.8
	nregex	2.1
	regex	1.6
Validate, split into CIGAR operations and capture groups	re	7.2
	nre	11.9
	nregex	8.8
	regex	4.9
Match, split by regular expression and capture named groups	re	n.a.
	nre	15.9
	nregex	12.3
	regex	5.3

Table notes:
library URL and versions used:
re, version Nim 1.4.8: `https://nim-lang.org/docs/re.html`,
nre, version 2.0.2: `https://nim-lang.org/docs/nre.html`,
nregex, version 0.0.4: `https://github.com/nitely/nregex`,
regex, version 0.16.2: `https://github.com/nitely/nim-regex`
