# Supplementary material for: TextFormats: Simplifying the definition and parsing of text formats in bioinformatics
Source: PLoS One. 2022 May 26;17(5):e0268910. doi: 10.1371/journal.pone.0268910 (PMC9135226; doi:10.1371/journal.pone.0268910)
Supplement: Attachment — Submitted filename: response-to-reviewers.pdf [file pone.0268910.s003.pdf]

Response to Reviewers of manuscript: TextFormats: simplifying the definition and parsing of text formats in bioinformatics

Giorgio Gonnella

February 17, 2022

1 Editor remarks

I would expect a comparative analysis as indicated by the reviewer.

The requested comparative analyses were implemented. The new Case study 4 (Pages 16–17, Lines 335–362) includes a comparative analysis with an existing Python library for parsing GFA2 and Case study 3 (Pages 14–16, Lines 299–333) now includes a comparative analysis with a newly implemented ad hoc parser when parsing the GFA2-inspired format defined there.

Adding another use case is also highly recommended.

The revised manuscript includes three additional use cases. Case study 4 (Pages 16–17, Lines 335–362) demonstrates the use of the library for rapid development of tools in Python to handle the sequence graph format GFA2. Case study 5 (Page 17, Lines 364–374) illustrates how TextFormats can be used for converting a table containing annotation data to a standard annotation file format (GFF3). Finally, Case study 6 (Page 18, Lines 376–401) shows how the library can be used for repairing a wrongly formatted GFA1 file output by another tool.

We note that you have provided additional information within the Acknowledgements Section that is not currently declared in your Funding Statement. Please note that funding information should not appear in the Ac-

knowledgments section or other areas of your manuscript. We will only publish funding information present in the Funding Statement section of the online submission form. Please include your amended statements within your cover letter; we will change the online submission form on your behalf.

The funding information was removed from the Acknowledgments section (Page 23, Lines 534–537). No change in the Funding Statement is necessary.

Please include captions for your Supporting Information files at the end of your manuscript, and update any in-text citations to match accordingly.

The captions for the Support Information files (S1 appendix and S2 appendix) are included at the end of the manuscript (Pages 22–23, Lines 526–532). The files were renamed to reflect the guidelines (upper case S).

2 Review 1

The choice of programming language is very peculiar. Nim is a language that is very rarely used in bioinformatics. With the growing popularity, flexibility, and user-friendliness of python-based tools and the availability of trusted legacy software written in C/C++/Java/Bash, it can be safely assumed that Nim as a language does not have much of a future in this domain.

Nim is indeed an unusual choice of programming language for this application domain - although software developed in the language has been published previously, such as hts-nim (Pedersen et al. 2018, Bioinformatics).

Assuming that Nim remains rarely used in bioinformatics also in the future, it can still be employed for implementing Python extension libraries. Nim is ultimately compiled to machine code (through C), thus offering a higher performance than Python, and allows more rapid development than C at the same time, with a syntax similar to Python itself. At the same time, besides in Python, the same Nim code can be used in C, C++ and potentially any other language with offers an interface to C code. These considerations were added to the Discussion (Pages 20–21, Lines 468–477).

Even though the authors have developed APIs and wrappers to support other languages in TextFormats, I believe trying to use this tool in other

programming languages for critical bioinformatics operation would prove unnecessarily tedious and time-consuming.

The use of TextFormats in Python is not more complex than using the library in Nim. The Python user can download the library using `pip` *and does not need to know much about Nim (and in many case not even to install it).*

S1 Appendix *includes examples of code in different programming languages (Python, Nim, Bash, C). The Python code of the example is almost identical to the Nim code and the interface is very intuitive. The C code is indeed slightly more complex, due to the nature of the C language itself (e.g. explicit memory management).*

To further demonstrate the usefulness of the library from Python, we now implemented several additional examples of use of the Python API, such as those discussed in Case study 4 *(Pages 16–17, Lines 335–362),* Case study 5 *(Page 17, Lines 364–374) and* Case study 6 *(Page 18, Lines 376–401).*

An analysis showing the compilation and run-time of TextFormats in different languages might prove otherwise

In Case study 1 (Pages 12–13, Lines 259–281), Case study 3 (Pages 14–16, Lines 299–333), Case study 4 (Pages 16–17, Lines 335–362) we now compare equivalent programs written in different languages (Nim, Python, C). The revised manuscript contains benchmark results which compare these implementations (Table 4). Using the library from Python, instead of from Nim, in equivalent programs resulted in a maximum overhead of about 10 %. These results were commented in the Discussion of the revised manuscript (Page 21, Lines 478–496).

Compilation of the library, when used in Python, is only necessary once, on installation (or not even then, when a pre-compiled binary distribution is compatible with the system). This required 41 seconds on the computer used for code development. All programs written in C must be compiled - a single time, before first use, but this was very fast as well. For example, compiling the C code used for the case studies required 11 seconds (Case study 1) and 10 seconds (Case study 3).

The compilation of the TFSL specification from YAML files is independent of the programming language choice and is always included in the running time mentioned in the manuscript. To quantify the TFSL compilation time alone, a new table (Table 2) was added, showing the time for compilation of the YAML specification for several included specifications.

The authors have mentioned other general-purpose standard data formats such as GFA2; however, the result and discussion do not portray any comparative analysis with such format. Such analysis is required, along with the ad-hoc format parsing

We added a new analysis as Case study 4 (Pages 16–17, Lines 335–362), which describes the development of Python tools for collecting statistics and validating the structure of a GFA2 file; thereby we compared the development of the tools using TextFormats to using an existing Python library (GfaPy) and commented the results in the Discussion (Page 19, Lines 414–421).

The GFA2 format is extensible, but aimed at a specific use case (sequence graphs). In Case study 3 (Pages 14–16, Lines 299–333) we used the same general structure of GFA2: a line-oriented tabular file, with different record types (each with his own field order, formatting and semantics) and optional information added using tags. Existing GFA2 libraries cannot handle these files, since they do not contain sequence graphs.

Thus, to provide a comparison with TextFormats, as suggested, we implemented ad hoc *format parsing tool in Python (Page 16, Lines 319–329) and compared it to the* TextFormats*-based implementation in the Discussion (Pages 19–20, Lines 430–442).*

Although the authors have mentioned two major applications of the tool by working with both alignment and sequence formats, they have omitted the tools applicability in another major use case, feature formats such as GFF, GTF.

The revised manuscript now includes an example application for annotations. In Case study 5 (Page 17, Lines 364–374), TextFormats is used for converting annotation data contained in a table into a valid standard-compliant GFF3 file.

Minor concerns - A visualization of the TFSL specification tree would clarify the specification representation clearly missing in the paper.

As new figure (Fig 1) was added to the revised manuscript. It contains an example of TFSL specification in YAML format and its visualization as a tree. See (Page 5, Lines 97–98).

Line number 3, binary format > formats Line 43, become > became I.e., e.g. etc. should be followed by a comma(,). Several minor grammatical mistakes

Thank you for pointing out this. The revised manuscript was now checked by a native speaker and some grammatical mistakes were corrected, including those mentioned above.

In conclusion, I believe that the software has novelty and can have a good prospect. However, as the premise of such a general-purpose framework is the user applications, the language should be one of current practice. Even then, I would recommend acceptance if the authors can provide substantial evidence regarding acceptable compilation and running time of the software in other languages through their developed APIs.

It is probable that most user will employ this library from Python. From the perspective of a Python user, the implementation language (Nim) does not really matter, as the library can be easily installed using the same mechanism (`pip install`) as other Python libraries. As mentioned above, code based on the library is very similar in both languages and there is a limited overhead (about 10%), compared to use within Nim.

As reported above, the compilation time of the library itself is not an issue. Compilation of the YAML specifications is also fast and is reported in the revised manuscript in the new Table 2, (Page 8, Line 178). The running times in different languages were summarized in Table 4, (Page 13, Line 282).
